# Efficacy and Safety of *Panax notoginseng* Saponin Therapy for Acute Intracerebral Hemorrhage, Meta-Analysis, and Mini Review of Potential Mechanisms of Action

**DOI:** 10.3389/fneur.2014.00274

**Published:** 2015-01-07

**Authors:** Dongying Xu, Ping Huang, Zhaosheng Yu, Daniel H. Xing, Shuai Ouyang, Guoqiang Xing

**Affiliations:** ^1^Faculty of Nursing, Guangxi University of Chinese Medicine, Nanning, China; ^2^Department of Oncology, Huanggang Hospital of Traditional Chinese Medicine, Huanggang, China; ^3^Thomas Wootton High School, Rockville, MD, USA; ^4^School of Business, University of Alberta, Edmonton, AB, Canada; ^5^Lotus Biotech.com LLC, Rockville, MD, USA

**Keywords:** notoginsenosides, botanical medicine, nutraceuticals, TBI and stroke recovery, randomized controlled clinical trials, hemostasis, anti-coagulation, pharmacological mechanisms

## Abstract

Intracranial/intracerebral hemorrhage (ICH) is a leading cause of death and disability in people with traumatic brain injury (TBI) and stroke. No proven drug is available for ICH. *Panax notoginseng* (total saponin extraction, PNS) is one of the most valuable herb medicines for stroke and cerebralvascular disorders in China. We searched for randomized controlled clinical trials (RCTs) involving PNS injection to treat cerebral hemorrhage for meta-analysis from various databases including the Chinese Stroke Trials Register, the trials register of the Cochrane Complementary Medicine Field, the Cochrane Central Register of Controlled Trials, MEDLINE, Chinese BioMedical disk, and China Doctorate/Master Dissertations Databases. The quality of the eligible trials was assessed by Jadad’s scale. Twenty (20) of the 24 identified randomized controlled trials matched the inclusive criteria including 984 ICH patients with PNS injection and 907 ICH patients with current treatment (CT). Compared to the CT groups, PNS-treated patients showed better outcomes in the effectiveness rate (ER), neurological deficit score, intracranial hematoma volume, intracerebral edema volume, Barthel index, the number of patients died, and incidence of adverse events. *Conclusion:* PNS injection is superior to CT for acute ICH. A review of the literature shows that PNS may exert multiple protective mechanisms against ICH-induced brain damage including hemostasis, anti-coagulation, anti-thromboembolism, cerebral vasodilation, invigorated blood dynamics, anti-inflammation, antioxidation, and anti-hyperglycemic effects. Since vitamin C and other brain cell activators (BCA) that are not considered common practice were also used as parts of the CT in several trials, potential PNS and BCA interactions could exist that may have made the effect of PNS therapy less or more impressive than by PNS therapy alone. Future PNS trials with and without the inclusion of such controversial BCAs as part of the CT could clarify the situation. As PNS has a long clinical track record in Asia, it could potentially become a therapy option to treat ICH in the US and Europe. Further clinical trials with better experimental design could determine the long-term effects of PNS treatment for TBI and stroke.

## Introduction

Traumatic brain injury (TBI) is a leading cause of death and disability in young people (1). Every year approximately 1.5 million people die and at least 10 million people are hospitalized after TBI (2). The incidence of TBI fatality and disability rates are higher in developing countries than in developed countries (3).

Secondary brain damage due to continued intracranial and intracerebral bleeding and hemorrhage swelling is a common cause of morbidity and mortality (4, 5). In one clinical trial, 56% of the patients with mild, moderate and severe TBI developed intracranial hemorrhage (6). Another study showed that 51% of TBI patients developed progressive intracranial/intracerebral hemorrhage (ICH), and hemorrhage expansion during the first 24–48 h after hospital admission (7). Prognostic studies have shown that ICH is associated with increased mortality and disability 6 months after injury (8, 9). One recent survey reported that TBI patients who developed ICH showed a 10-fold increase in stroke incidence 3 months after the injury when compared to TBI patients without ICH (10).

Acute intracerebral hemorrhage (AICH) accounts for only about 10% of the people with stroke, and is the most lethal form of stroke compared to the ischemic stroke. Thus, ICH is among the most devastating disorders and a leading cause of disability and mortality of people with severe stroke, hypertension, and TBI (11). During the last decade, the incidence of ICH has increased steadily in Asian countries (12) and it accounts for ~20% and ~10% of strokes in low-middle and high income countries, respectively (13).

So far, few proven therapies exist for ICH. Hematoma expansion, perihematomal edema with increased intracranial pressure, intraventricular extension of hemorrhage with hydrocephalus, seizures, venous thrombotic events, hyperglycemia, increased blood pressure, fever, and infections are among the complications of ICH as recently reviewed by Balami and Buchan (14). Current treatment of ICH is supportive and life-sustaining rather than a complete cure that aims to limit secondary brain damage and associated complications (15, 16). Considering the very limited therapeutic options for patients with ICH, recent studies suggest that evidence-based alternative and complimentary medicines could be effective in reducing the adverse effects early in the course of ICH and in improving its prognosis as found in the treatment of cerebral ischemia (17).

**Figure 1:**
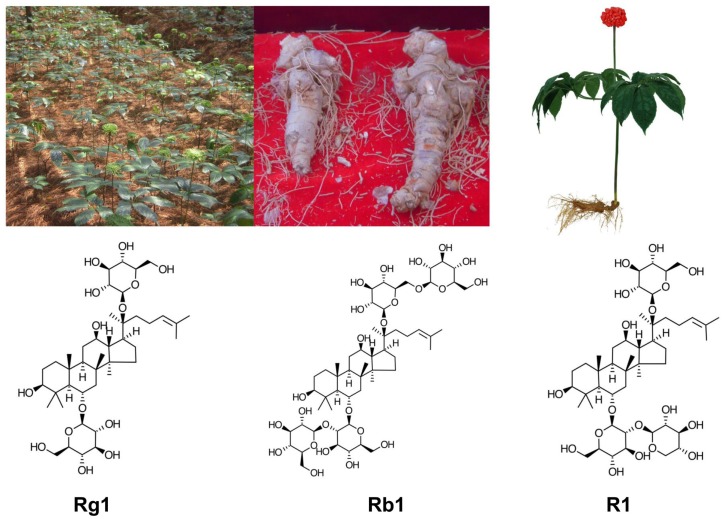
***Panax notoginseng* (*P. notoginseng*, Sanqi): clockwise from top left: in cultivation, the roots, artistic drawing a Sanqi plant, the main chemical ingredients of PNC: notoginsenoside R1, ginsenosides Rb1, and Rg1**.

*Panax notoginseng* [(Burk.) F.H. Chen] (also called Sanqi in Chinese), is one of the most valuable Chinese herbal medicine. *P. notoginseng* is a perennial plant, mainly grown in the high mountain areas of Southwest China. Its roots are harvested after 3–5 years of growth (Figure 1). *P. notoginseng* has numerous hematological and pharmacological effects, which include regulation of platelet aggregation and platelet free calcium levels, reducing blood viscosity, improving local blood supply and circulation to end stasis, cerebral vasodilation, analgesic, hypolipidemic, hemostatic, anti-edema, anti-hyperglycemia, antioxidation, anti-inflammation, and anti-apoptosis (18, 19). *P. notoginseng* saponins extract (PNS) ameliorate learning and memory deficits in animals (20–26), probably by inhibiting oxidative stress and apoptosis and by stimulating neurogenesis (21, 22, 27–33). PNS is effective against ICH, transient focal ischemia, and cerebral infarction probably in part through improved brain blood circulation and energy metabolism (34–38). *P. notoginseng* has been used alone and as a key tonic ingredient in many other patent Chinese medicine for treatment of a variety of health conditions and has been proved to be effective in animal models of cerebral ischemia/reperfusion injury, arterial thrombosis, cardiovascular disorders, Alzheimer disease, diabetes and obesity, erectile dysfunction, neurodegeneration, neuroinflammation, oxidative stress, neurotoxicity, organ injury, and cancer (38–47).

The ability of notoginseng to normalize hemorheological parameters is due to the presence of multiple active compounds including different ginsenosides and notoginsenosides, some of which appear to have similar yet differential effects. Ginsenosides Rg1 and Rb1, and notoginsenoside R1 are the main active ingredients present in high concentrations in PNS, which contains more than 20 different ginsenosides and notoginsenosides (48–55). Purified and patented PNS under different trade names, i.e., Xuesaitong (51, 56–59), Xueshuantong (60), and Lulutong have been approved for treatment of stroke and other cerebral disorders in China. Intravenous injection of PNS has been developed for critical care because orally administered PNS has a low permeability, poor intestinal absorption, and bioavailability (51, 61, 62) due to the relative large sugar molecular mass (>500 Da), high molecular flexibility, high hydrogen binding capacity, and low lipophilicity. *In vivo* studies showed marked variability of Rb1 bioavailability among different administration routes to rats: i.v. (intravenous) (100%) > p.v. (portal venous) (59.49%) > i.d. (intra-duodenal) (2.46%) > p.o. (peroral) (0.64%) (63). After absorption, PNS has a long residual time, but individual ginsenosides and notoginsenosides vary in their elimination rate. For example, the half-life is much longer and peak concentration higher for ginsenoside Rb1 than that for ginsenoside Rg1 and notoginsenoside R1, due to the slower clearance and longer residence time of Rb1 (61, 63–67). In fact, ginsenoside Rb1 is considered a pharmacokinetic marker of PNS (67).

Multiple clinical studies have been conducted in China in recent years to explore the use of PNS injection in the treatment of cerebral hemorrhage and ICH. These studies have yet to be systematically evaluated for the efficacy and safety to provide evidence and guidance for further clinical application of PNS for cerebral hemorrhage. Because many of these trials were reported in un-indexed Chinese medical journals, the international research community may not have access to these findings. In this study, we reviewed randomized controlled clinical trials (RCTs) published in Chinese journals that involved PNS injection treatment for ICH. The results suggest that PNS injection has a wide range of different protective mechanisms and is a better treatment than current treatments for ICH.

## Materials and Methods

### Inclusive and exclusive criteria

Only RCTs that included a comparison of the efficacy and safety of *P. notoginseng* saponin (PNS) injection treatment with that of current treatment (CT) in patients with AICH resulted from hypertension or wind stroke were included. The diagnostic criteria of acute ICH in trials were in accordance with the criteria of diagnosis of various types of cerebrovascular disease updated at the 4th Annual Conferences of Chinese Society of Neurology (68): subjects should not suffer from secondary AICH or other diseases such as hematologic diseases, intracranial aneurysms, intracranial tumors, cerebral arteries, venous malformations, severe comas, and cardiac, hepatic, or renal diseases. There was no restriction on race, but the majority of patients were thought to be of the Chinese Han ethnic group. The PNS intervention is defined as intravenous drip of the commercially available PNS preparations that are approved by Chinese Food and Drug Administration (CFDA) for clinical use alone, or in combination with other routine therapies for subjects in the treatment groups. The control ICH subjects received current treatment other than PNS treatment. The outcome measures included the effectiveness rate (ER), neurological deficit score (NDS), intracerebral hematoma volume (IHV), intracerebral edema volume (IEV), Barthel index (BI), and number of patients died (NDP), as well as incidence of adverse events after treatments with PNS or CT, respectively. The BI is an interviewer-based disability profile scale developed by D.W. Barthel in 1965 to assess physical functions, specifically self-care abilities and ambulation (e.g., stair climbing) in 10 areas, including bowel and bladder control. The patient is scored from 0 to 15 points in various categories, depending on his or her need for help, such as in feeding, bathing, dressing, and walking.

### Search strategy

The search strategy was developed by modifying the reported strategies used for herbal medicines in a Cochrane review (69). We retrieved the literatures of relevant clinical trials by electronic searching and by hand searching, regardless of language or publication status. Many electronic databases were searched, including the Chinese Stroke Trials Register, the trials register of the Cochrane Complementary Medicine Field, the Cochrane Central Register of Controlled Trials, MEDLINE, CINAHL, AMED, Chinese BioMedical disk, Wanfang Chinese Scientific Journal Database, VIP, China National Knowledge Infrastructure, Traditional Chinese Medicine Database, Chinese Medical Current Contents, China Doctorate/Master Dissertations Full-Text Databases, and China Proceedings of Conference Databases. The reference lists of retrieved papers were further scanned for any possible titles matching the inclusive criteria. A hand search with an emphasis on relevant journals pertaining to stroke, senile disease, neurology, complementary, and alternative medicine was carried out to explore entities matching the inclusive criteria among periodicals, journals, and symposium abstracts found in libraries of Guangxi Chinese Medical University (date of last search: December, 2013).

### Data extraction

Full-text articles of each potential eligible trial were retrieved and assessed by two independent reviewers (Dongying Xu and Ping Huang) to determine if the articles should be recruited and further analyzed according to the inclusive and exclusive criteria. Missing information was sought by contacting the article authors. A data abstraction form was used to summarize key information from included trials, and key information was extracted by one reviewer and confirmed by the other. Any disagreements were resolved by discussion.

### Data analysis

The meta-analysis was carried out by using Revman 5.3 software (Cochrane Collaboration) to combine and analyze the data from the individual trials. The statistical validity of combining various trials was assessed by examining the homogeneity of outcomes from trials using a *Q*-test (Mantel–Haenszel Chi-square test). The results of the combined trials were calculated with random or fixed-effect models. The measurements of each category’s data were evaluated by a weighted mean difference (WMD) or odd ratio (OR), and by 95% confidence intervals (95% CI). The methodological quality of all included trials was assessed by Jadad’s scale that evaluated randomization, double blinding, and dropout rate of the trials by ranking them with 1–5 points. The trials that scored with 1 or 2 points were considered low-quality trials, while those that scored with 3–5 points were considered high-quality trials (70).

## Results

### Excluded and included trial

The literature search yielded a total of 24 RCTs conducted in China that treated acute intracerebral hemorrhagic patients with intravenous drip of PNS. However, four of these trials were excluded as they did not match the inclusive criteria. Specifically, they are (1) no-comparison made between PNS and RT in trials (two trials) and (2) no or unclear measurement report in trials (two trials). Therefore, only 20 trials published in Chinese medical journals, with 1,891 ICH patients that met the study criteria, were included for analysis (71–90).

The characteristics of the patients are shown in Table 1. The trials’ size varied from 24 to 200 participants, with an average of 46 patients per trial. All of the patients are adults ranged from 24 to 92 years old with more males than females included (62% males vs. 38% females). Hemorrhage/ICH duration was reported in 13 trials, ranging from 4 h to 20 days. The main causes of intracerebral hemorrhage were wind stroke (15 trials) and hypertension (2 trials) (Table 1).

**Table 1:** **Baseline characteristics of each trial used in the meta-analysis**.

Authors	Sample size (F/M) Average age (range)	Disease duration	Cause
Guo et al. (90)	PNS: *n* = 100 (40/60) 57.4 (30–70)	≥ 3D	WS
	CT: *n* = 100 (38/62) 57.2 (31–69.5)		WS
He et al. (89)	PNS: *n* = 12 (5/7) 69.20 ± 14.32	≤ 48H	WS
	CT: *n* = 12 (4/8) 67.22 ± 13.83		WS
Xu and Dong (72)	PNS: *n* = 42 (17/25) 60.63 (33–80)	NR	WS
	RT: *n* = 40 (17/23) 58.30 (34–79)		WS
Li et al. (88)	A:PNS: *n* = 60 (25/35) (24–78)	< 24H	WS
	B:PNS: *n* = 55 (22/33) (31–75)		WS
	C:CT: *n* = 60 (26/34) (40–82)		WS
Li and Yang (73)	PNS: *n* = 48 (13/35) 57.2 ± 9.6 (45–75)	> 20D	WS
	CT: *n* = 44 (12/32) 56.8 ± 9.4 (43–74)		WS
Tian et al. (74)	PNS: *n* = 36 (16/20) 60.32 ± 5.14	NR	WS
	CT: *n* = 30 (16/14) 58.41 ± 6.33		WS
Li and Sun (75)	PNS: *n* = 29 (9/20) 58.5 ± 10.8 (31–82)	NR	NR
	CT: *n* = 31 (9/22) 57.5 ± 11.2 (33–81)		NR
Xie et al. (76)	PNS: *n* = 24 (6/18) 61.1 (34–89)	NR	NR
	CT: *n* = 22 (6/16) 61.2 (35–92)		NR
Chen et al. (77)	PNS: *n* = 22 (7/15) N/A	4H-12H	WS
	CT: *n* = 21 (7/14) N/A		WS
Dong and Wang (78)	PNS: *n* = 40 (16/24) 60.63 (33–80)	NR	HYTN
	CT: *n* = 38 (16/22) 58.3 (34–79)		HYTN
Zhang et al. (71)	PNS: *n* = 65 (N/A) 57.6 (35–69)	NR	WS
	CT: *n* = 65 (N/A) 58.2 (36–70)		WS
Zhou et al. (79)	PNS: *n* = 70 (24/46) 56.8 ± 10.4 (35–78)	≤ 7D	WS
	CT: *n* = 70 (27/43) 55.6 ± 10.1 (33–79)		WS
Zheng (80)	PNS: *n* = 22 (N/A) N/A	< 72H	N/A
	CT: *n* = 19 (N/A) N/A		N/A
Tang et al. (81)	PNS:*n* = 63 (25/38) 62.2 ± 14.6	3rd	WS
	CT: *n* = 63 (23/40) 62.5 ± 15.2		WS
Song (82)	PNS: *n* = 39 (12/27) N/A	≤ 48H	WS
	CT: *n* = 39 (15/24) N/A		WS
Ding et al. (83)	PNS: *n* = 15 (6/9) 55.1 (35–74)	< 48H	WS
	CT: *n* = 17 (4/13) 57.7 (44–72)		WS
Yuan et al. (84)	PNS: *n* = 67 (26/41) 59.2 (39–75)	NR	WS
	CT: *n* = 66 (27/39) 61.3 (41–75)		WS
Ding and Geng (85)	PNS: *n* = 86 (34/52) 60.2 ± 8.5	≥ 7D	HYTN
	CT: *n* = 86 (37/49) 61.4 ± 8.2		HYTN
Gao et al. (86)	PNS: *n* = 28 (12/16) 65.68 ± 9.55	≤ 24H	WS
	CT: *n* = 24 (11/13) 65.88 ± 9.47		WS
Chen (87)	PNS: *n* = 61 (23/38) 60.7 ± 7.7 (38–74)	≥ 7D	WS
	CT: *n* = 60 (19/41) 61.3 ± 8.0 (36–75)		WS

*CT, current treatment; D, day; H, hour; HYTN, hypertension; N/A, not applicable; NR, not reported; PNS, *Panax notoginseng* saponin; WS, wind stroke [Stroke including hemorrhagic cerebrovascular accident (CVA), ischemic infarction CVA and subarachnoid hemorrhage, etc. is often collectively referred to as wind stroke in traditional Chinese medicine because of its sudden and acute onset, sudden loss of consciousness with unilateral weakness, numbness, paralysis and dysphasia with or without experiencing unconsciousness, multiple symptoms and rapid alterations in manifestations that are similar to the natural characteristics of wind, which is changing rapidly]*.

The classification of the patients (sites of bleeding) is shown in Table 2. Of these, 984 ICH patients received PNS injection treatments that lasted between 10 and 70 days, and 907 patients received current treatments (Table 2). Of the PNS injection treatments, 6 trials used Xuesaitong PNS freeze-dry powder injections (72, 73, 77–79, 85), 7 trials used Xuesaitong injections (74, 75, 84, 86–88, 90), 5 trials used Xueshuantong injections (76, 80, 81, 83, 89), one trial used Lulutong injections (71), and one trial used Sanqi Zaogan injection powder (82). For the CT control groups, dehydration, control of intracranial pressure, anti-hypertensive treatment, symptomatic treatment, neurotrophy medicine, and brain cell activators (BCA) were used. Mannitol, glycerol, and/or ructose injection was used for dehydration (Table 2).

**Table 2:** **PNS treatment information of the 20 trials included in the meta-analyses**.

Authors	Interventions (sample size) Dosage	Durations	Observations	Adverse incidences (%)
Guo et al. (90)	PNS(*n* = 100): XST Inj 200 mg/day	3 weeks	ER	NO
	CT(*n* = 100): MNT,ST			NR
He et al. (89)	PNS(*n* = 12): XSHT Inj 140 mg/day	2 weeks	IHV,IEV	NR
	CT(*n* = 12): MNT,VC,KCI			
Xu and Dong (72)	PNS(*n* = 42): XST FDP 400 mg/day	4 weeks	ER,NDP	NR
	CT(*n* = 40): DH,ICP,ST			
Li et al. (88)	PNS(*n* = 55): XST Inj 600 mg/day	2 weeks	ER,NDP,IHV	Sr (5%)
	CT(*n* = 60): AHT,CICP,ST			NO
Li and Yang (73)	PNS(*n* = 48): XST FDPI 400 mg/day)	2 weeks	ER,NDS	NO
	CT(*n* = 44): CAS			NR
Tian et al. (74)	PNS(*n* = 36): XST Inj 200 mg/day	6 weeks	ER,NDP,NDS,IHV	NR
	CT(*n* = 30): MNT,FRS,ST			
Li and Sun (75)	PNS(*n* = 29): XST Inj 500 mg/day	4 weeks	ER	NR
	CT(*n* = 31): DH,NTM,BCA,ST			
Xie et al. (76)	PNS(*n* = 24): XSHT Inj 400 mg/day	10 weeks	ER,BI	NO
	CT(*n* = 22): DH,CICP,ST			
Chen et al. (77)	PNS(*n* = 22): XST FDPI 800 mg/day	2 weeks	NDS	NO
	CT(*n* = 21): DH,CICP,AHT,ST			
Dong and Wang (78)	PNS(*n* = 40): XST FDPI 400 mg/day	2 weeks	ER,NDP	NR
Zhang et al. (71)	CT(*n* = 38): DH,CICP,ST	2 weeks	ER	NO
	PNS(*n* = 65): LLT Inj 250 mg/day	
	CT(*n* = 65): MNT, MGSO_4_,KCL,			
	INS, Aceglutamide Inj			
Zhou et al. (79)	PNS(*n* = 70): XST FDPI 400 mg/day	2 weeks	ER,NDS	NR
	CT(*n* = 70): CICP,MNT,FRS Inj,			
	GFI,BCA,AHT,ST			
Zheng (80)	PNS(*n* = 22): XSHT Inj 300 mg/day	3 weeks	NDS	NR
	CT(*n* = 19): ST			
Tang et al. (81)	PNS(*n* = 63): XSHT Inj 300 mg/day	3 weeks	IHV	NR
	CT(*n* = 63): MNT,VC,ST			
Song (82)	PNS(*n* = 39): XSHT Inj powder 450 mg/day	4 weeks	IHV,IEV	NR
	CT(*n* = 39): CICP, AHT,ST			
Ding HY 2008 (83)	PNS(*n* = 15): XST FDPI 200 mg/day	2 weeks	BI	NR
	CT(*n* = 17):AHT, MNT,CICP, ST			
Yuan HY 2008 (84)	PNS(*n* = 67): XST Inj 750 mg/day	2 weeks	ER,NDP	NO
	CT(*n* = 66): MNT,GFI			NR
Ding and Geng (85)	PNS(*n* = 86): XSHT Inj 175 mg/day	3 weeks	ER,IHV,IEV	NO
	CT(*n* = 86): CICP, AHT,ST			
Gao HY 2008 (86)	PNS(*n* = 28): XST Inj 250 mg/day	2 weeks	NDS	NR
	CT(*n* = 24): MNT,CICP, AHT,ST			
Chen (87)	PNS(*n* = 61): SQZG 350 mg/day	2 weeks	ER,NDP,IHV	NR
	CT(*n* = 60): CICP, AHT,ST			

*AHT, anti-hypertensive treatment; BCA, brain cell activators; BI, Barthel index; CAS, citicoline sodium; CICP, control of intracerebral pressure; DH, dehydration; ER, effectiveness rate; DPI, freeze-dry powder injector; FRS, furosemide; GFI, glycerol and fructose injection; IEV, intracerebral edema volume; IHV, intracerebral hematoma volume; Inj, injection; INS, insulin; KCI, potassium chloride; MGSO_4_, magnesium sulfate; MNT, mannitol; NDP, number of death patients; NDS, neurological deficit score; NR, not reported; NTM, neurotrophy medicine; PNS, *Panax notoginseng* saponin; CT, current treatment; SQZG, sanqi zaogan; Sr, skin rashes; ST, symptomatic treatment; VC, vitamin C; XSHT, Xue Shuan Tong; XST, Xue Sai Tong (Xuesaitong)*.

### Outcome measurement

For evaluating the therapeutic and adverse effects of the PNS treatment and the control groups, the outcome assessment of this study was focused on the ER, NDS, intracerebral hematoma volume (IHV), IEV, BI, and the NDP. The incidence of adverse events after treatments was also evaluated. Thirteen trials reported the number of improved patients. Six trials reported NDS. Eight trials reported intracerebral hematoma volume. Three trials reported intracranial edema volume. Two trials reported BI, and six trials reported the NDP.

### Meta-analysis on PNS efficacy

#### Sites of bleeding

There were 15 trials involving 687 ICH patients in the PNS group and 583 patients in the CT group (total of 1,270 ICH patients) that provided detailed descriptions of the bleeding sites including (unilateral and/or bilateral) the basal ganglia, external capsule, internal capsule, frontal lobe, medial occipital lobe, arietal lobe, cerebellum, brainstem, ventricles, and supratentorial hemorrhage (Table 3). Among them, the basal ganglia region was the most common site of hemorrhage, accounting for 45.85% (315/687) of patients in the PNS group, and 41.51% (242/583) of patients in the CT group.

**Table 3:** **Detailed information of the hemorrhage sites of the 20 trials included in the meta-analyses**.

Authors	Sample size	Intervention time	Hematoma volume	Sites of bleeding (cases)
Guo et al. (90)	PNS(*n* = 100)	3rd D	10–60 ml	BGR (40),thalamus (20),lobar (33),cerebellar (7)
	CT(*n* = 100)			BGR (42),thalamus (16),lobar (36),cerebellar (6)
He et al. (89)	PNS(n = 12)	5th D	10–30 ml	UBG (12)
	CT(*n* = 12)			UBG (12)
Xu and Dong (72)	PNS(*n* = 42)	10th–15th D	<30 ml, 30–50 ml, >50 ml	Putamen (18),thalamus (9), lobar (6),brainstem (6),cerebellar (3)
	CT(*n* = 40)			Putamen (17),thalamus (8), lobar (7),brainstem (6),cerebellar (2)
Li et al. (88)	A:PNS(*n* = 60)	≤ 48H, ≥ 7 D	<42 ml	A:BGR (46),FL (3),EC (1),IC (2),brainstem (3),
				Ventricle (2), thalamus (1),cerebellar (2)
	B:PNS(*n* = 55)			B:BGR (43),FL (2),EC (2),IC (3),brainstem (2),
				Ventricle (1), cerebellar (2)
	C:CT(*n* = 60)			C:BGR (45),FL (2),EC (2),IC (3),brainstem (3),
				Ventricle (2), thalamus (1),cerebellar (2)
Li and Yang (73)	PNS(*n* = 48)	20th–22nd D	<40 ml	Lobar (12), EC or BG region (21), IC (9), ventricle (2), cerebellar (4)
	CT(*n* = 44)			Lobar (10), EC or BG region (22), IC (8), ventricle (1), cerebellar (3)
Tian et al. (74)	PNS(*n* = 36)	≤ 48H	≤30 ml	BGR (16), thalamus (11), lobar (9)
	CT(*n* = 30)			BGR (13), thalamus (10), lobar (7)
Li and Sun (75)	PNS(*n* = 29)	1st–15th D	6–58 ml	MBG (15), BGR (10), parietal lobe (2), FL (1),temporal (1)
	CT(*n* = 31)			NR
Xie et al. (76)	PNS(*n* = 24)	3rd W	SH ≤ 30 ml, cerebellar ≤ 15 ml,	SH (20) cerebellar (3), brainstem (1)
			Brainstem ≤ 5 ml	
	CT(*n* = 22)			SH (17) cerebellar (4), brainstem (1)
Chen et al. (77)	PNS(*n* = 22)	2nd W	≤ 25 ml	SH (22)
	CT(*n* = 21)			SH (21)
Dong and Wang (78)	PNS(*n* = 40)	10th–15th D	<30 ml, 30–50 ml, >50 ml	Putamen (18),thalamus (8),lobar (5),brainstem (6),cerebellar (3)
	CT(*n* = 38)			Putamen (17),thalamus (8),lobar (6),brainstem (5),cerebellar (2)
Zhang et al. (71)	PNS(*n* = 65)	3rd–8th D	30–50 ml	NR
	CT(*n* = 65)			
Zhou et al. (79)	PNS(*n* = 70)	4th–7th D	6–40 ml	BG (48), lobar (17), cerebellar (5)
	CT(*n* = 70)			BG (44), lobar (20), cerebellar (6)
Zheng (80)	PNS(*n* = 22)	≤ 48H	<30 ml	NR
	CT(*n* = 19)			
Tang et al. (81)	PNS(*n* = 63)	≥ 3 D	10–40 ml	NR
	CT(*n* = 63)			
Song (82)	PNS(*n* = 39)	≤ 48H	Low to medium	SH 39
	CT(*n* = 39)			SH 39
Ding et al. (83)	PNS(*n* = 15)	≤ 48H	≤30 ml	BGR (15)
	CT(*n* = 17)			BGR (17)
Yuan et al. (84)	PNS(*n* = 67)	≤ 48H	10–30 ml	BGR (49), temporal (5),FL (7), OL (5), cerebellar (<5 ml) (1)
	CT(*n* = 66)			BGR (47), temporal (6), FL (7), OL (3), cerebellar (<5 ml) (3)
Ding and Geng (85)	PNS(*n* = 86)	≥ 7 D	Low to medium	NR
	CT(*n* = 86)			
Gao et al. (86)	PNS(*n* = 28)	≤ 48H	<30 ml	Thalamus (7),putamen (15),caudate nucleus (2),lobar (4)
	CT(*n* = 24)			Thalamus (7),putamen (13),caudate nucleus (2),lobar (2)
Chen (87)	PNS(*n* = 61)	≥ 7 D	≤ 40 ml	NR
	CT(*n* = 60)			

*BG, basal ganglia; BGR, basal ganglia region; CT, current treatment; EC, external capsule; FL, frontal lobe; IC, internal capsule; MBG, medial basal ganglia; OL, occipital lobe; PNS, *Panax notoginseng* saponin; PL, parietal lobe; SH, supratentorial hemorrhage; UBG, unilateral basal ganglia*.

#### Effectiveness rate

**Figure 2:**
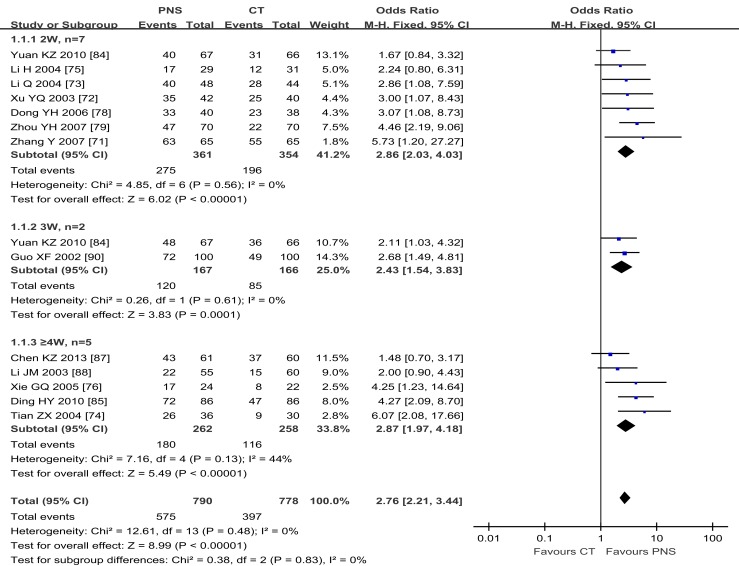
**Meta-analyses of the effectiveness rate (ER), comparing the therapeutic effects of PNS injection with that of the current treatment (CT)**. There is an overall significant statistical difference in ER between the PNS and CT groups (*P* < 0.00001). And the differences in ER between the two treatment groups were significant at 2, 3, and 4 weeks (*P* < 0.00001, each) after PNS treatment. PNS, *Panax notoginseng* saponin; CT, Current treatment.

By using the fixed-effect model, Figure 2 shows the results of meta-analyses on the ER, comparing the therapeutic effect of PNS injection with that of the CT. A total of 13 trials reported the effect rate, which was categorized into three subgroups by the evaluating time: (1) 7 trials assessed the ER at the end of 2 weeks of treatment, (2) 2 trials assessed the rate at the end of 3 weeks, and (3) 5 trials assessed the rate at the end of 4 weeks. There was no significant heterogeneity among these three subgroups (*P* = 0.47). The total overall effect showed significant statistical difference in ER between the PNS and CT groups (OR = 2.70; 95% CI = 2.16, 3.38; *P* < 0.00001). There were significant differences in ER between PNS and CT groups assessed at 2 weeks (OR = 2.73; 95% CI = 1.92, 3.88; *P* < 0.00001), 3 weeks (OR = 2.43; 95% CI = 1.54, 3.83; *P* = 0.0001), and 4 weeks (OR = 2.87; 95% CI = 1.97, 4.18; *P* < 0.00001) after the start of treatment. No significant heterogeneity was presented in the analyses of the data (*P* = 0.55, 0.61, and 0.13) in the three subgroups, respectively. Thus, ICH patients with PNS treatment showed a better therapeutic ER than those in the CT group.

#### Neurological deficit score

**Figure 3:**
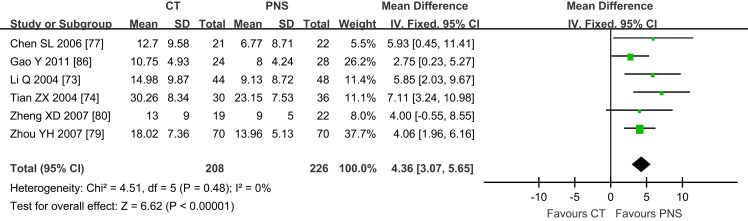
**Meta-analyses of the neurological deficit score (NDS)**. The overall effect showed significant statistical difference in NDS between the PNS and CT groups (MD = 4.36; 95% CI = 3.07, 5.65; *P* < 0.00001). No heterogeneity (*P* = 0.48) was found in NDS (6 trials). PNS, *Panax notoginseng* saponin; CT, Current treatment.

Neurological deficit score is an important index for the diagnosis of symptom severity and functional recovery of the patients (91). Six of the selected trials reported NDS in this study, with three of them showing NDS (74, 77, 86) at 7, 15, 21, 28, and 30 days after PNS treatment (Figure 3). No significant differences in NDS were found between the PNS and CT groups at 7 (77) and 15 days (86). The results of 21 days are controversial, since one trial showed no difference between the PNS and CT groups (74), whereas the other showed a better NDS in the PNS group than in the CT group (77). The NDS was significantly lower in PNS group than that in CT group at 28 days (86) and 40 days (74) after the treatment (*P* < 0.05,*P* < 0.01, respectively). No heterogeneity (*P* = 0.48) was found in the NDS. When all data were combined, the results showed significantly reduced NDS in PNS-treated ICH patients than in ICH patients of the CT group (MD = 4.36; 95% CI = 3.07, 5.65; *P* < 0.00001) (Figure 3).

#### Intracerebral hematoma volume

**Figure 4:**
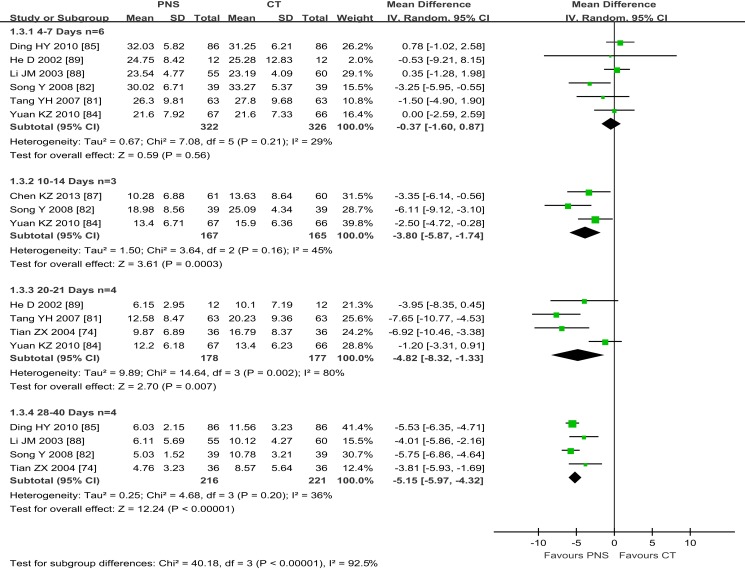
**Meta-analyses of intracerebral hematoma volume (IHV) among four subgroups with different time length of PNS treatment: (1) the subtotal group of 6 trials with 4–7 days of PNS treatment (Ding HY, He D, Li JM, Song Y, Tang YH, Yuan K) and, (2) the subgroup of 3 trials with 10–14 days of treatment (Chen KZ, Song Y, Yuan KZ) showed no significant differences in IHV between PNS and CT groups; (3) the subgroup of 4 trials with 20–21 days of PNS treatment (He D, Tang YH, Yuan KZ, Tian ZX) and, (4) the subgroup of 3 trials with 28–40 days of treatment [40, 78, 108,] (Ding YH, Li JM, Song Y, Tian ZX) showed significant improvement in IHV in the PNS group than in the CT group (*P*<0.00001, each)**. PNS, *Panax notoginseng* saponin; CT, Current treatment.

Of the 8 trials that reported IHV, 6 trials showed no differences in IHV between the PNS and CT groups after 4–7 days of treatment (82, 84, 85, 88, 89) (MD = −0.37; 95% CI = −1.60, 0.87; *P* = 0.58) (Figure 4). Three trials (82, 84, 87) showed significant improvement in IHV in the PNS group (about 25% less IHV) than in the CT group at 10–14 days after the treatment (MD = −3.80; 95% CI = −5.87, −1.74; *P* = 0.0003). Four trials showed significantly smaller IHV values in the PNS group (about 40% less) than in the CT group at 20–21 days after the treatment (74, 81, 84, 89) (MD = −4.82; 95% CI = −8.32, −1.33; *P* = 0.007). Another 4 trials showed significantly smaller IHV values in the PNS group (about 50% less) than in the CT group at 28–40 days after the treatment (74, 82, 85, 88) (MD = −5.15; 95% CI = −5.98, −4.33; *P* < 0.00001). These results show a time-dependent effect of PNS treatment on IHC, i.e., significant improvement in IHV occurs after 21 days or more of PNS treatment, but not in the 1st week of treatment (10–14 days, MD = −3.8;20–21 days, MD = −4.82; 28–40 days, MD = −5.15).

#### Intracerebral edema volume

**Figure 5:**
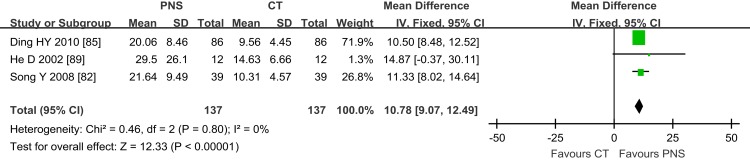
**Results of meta-analyses on intracerebral edema volume (IEV) involving 3 trials**. There was statistical difference in IEV values between PNS and CT groups (MD = 10.78; 95% CI = 9.07, 12.49; *P* < 0.00001). PNS, *Panax notoginseng* saponin; CT, Current treatment.

Figure 5 shows the results of meta-analyses on IEV. Only three trials (82, 85, 89) assessed IEV in ICH patients with PNS/CT treatment 7 days after ICH onset. Two of the trials with a duration of 3–4 weeks showed significant differences in IEV between the PNS and CT groups (65, 68). The other trial showed no significant difference between the two groups after 14 days of PNS injection due to a small sample size (89) (MD = 14.87; 95% CI = −0.37, 30.11). Analysis of the combined data showed significant statistical difference in IEV values between the PNS and CT groups (MD = 10.78; 95% CI = 9.07, 12.49; *P* < 0.00001) (Figure 5). On average, PNS treatment reduced IEV value by about 50%.

#### Barthel index

**Figure 6:**
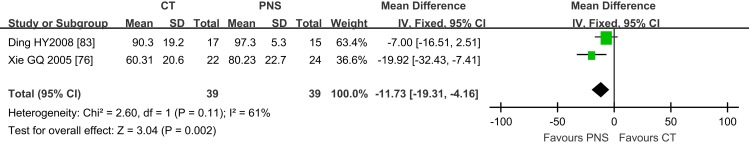
**Results of meta-analyses on Barthel index (BI) (2 trials)**. PNS treatment significantly increased BI compared to RT (MD = -11.73; 95% CI = -19.31, -4.16; *P* = 0.002). PNS, *Panax notoginseng* saponin; CT, Current treatment.

The BI is a measure of functional disability and represents the current quality of life (92). Only 2 trials of the 20 trials examined in this study reported BI (76, 83). The results showed that PNS treatment significantly increased BI when compared to the CT (MD = -11.73; 95% CI = -19.31, -4.16; *P* = 0.002) (Figure 6). One trial involved 10 weeks of PNS treatment after ICH and the result showed no significant difference in BI at 14 days after PNS treatment (*P* > 0.05), but significant differences were observed at 28 days (*P* < 0.05) and 90 days after the treatment (*P* < 0.01), suggesting that a relatively long course of PNS treatment is necessary for significant improvement in functional recovery (76).

#### Mortality rate

**Figure 7:**
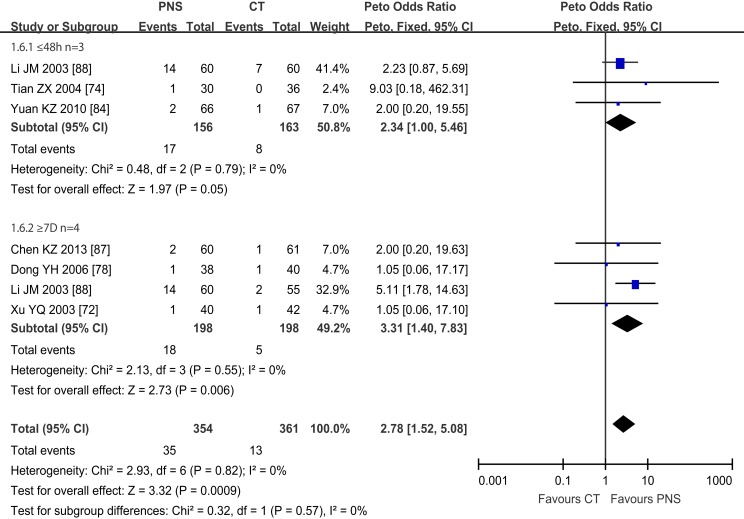
**Meta-analyses of number of patients died (NDP) in 6 trials**. The total number of patients died (NDP) was 48 out of 715 (6.7%). The mortality of ICH patients was significantly lower in PNS group (13/361, 3.6%) than in CT group (35/354, 9.9%) (Peto OR = 2.78; 95% CI = 1.52, 5.08; *P* = 0.0009). The trials were divided into two subgroups based on the intervention time of PNS: (1) 3 trials with PNS treatment started within 48 h after ICH onset (Li JM, Tian ZX, Yuan KZ), (2) 4 trials with PNS treatment started 7 days from ICH onset [25, 41, 78, 131] (Chen KZ, Dong YH, Li JM, Xu YQ), One trial (LI JM) had trials started both within 48 h and 7 days after ICH onset. All trials assessed the mortality at the end of treatment course. Both PNS treatment regimes significantly reduced the mortality of ICH patients compared to the CT groups (*P* = 0.05; *P* = 0.006, respectively). PNS, *Panax notoginseng* saponin; CT, Current treatment.

Figure 7 shows the results of meta-analyses on the NDP. Six trials reported mortality data, showing that 48 of the 715 patients across the six trials died (6.7%). The mortality of ICH patients was significantly lower in the PNS group (13/361, or 3.6%) than in the CT group (35/354, or 9.9%) (Peto OR = 2.78; 95% CI = 1.52, 5.08; *P* = 0.0009). The trials were further divided into two subgroups depending on the time of PNS intervention: (1) three trials that started PNS treatment within 48 h of ICH onset (74, 84, 88) and (2) four trials that started PNS treatment at or after 7 days of ICH onset (72, 78, 87). One trial evaluated PNS intervention at both 48 h and 7 days after ICH onset (88). All trials assessed the mortality at the end of the treatment course. The duration of PNS treatment was 4 (72) and 6 (74) weeks, respectively, for 2 of the trials, and was 2 weeks for the other trials. The results showed significant reduction in the mortality of ICH patients treated with PNS within 48 h of ICH onset (Peto OR = 3.31; 95% CI = 1.40, 7.83; *P* = 0.006), but no significant differences between the PNS and CT groups when PNS treatment started more than 48 h after ICH onset (Peto OR = 2.34; 95% CI = 1.00, 5.46; *P* = 0.05), suggesting that early PNS intervention is critical in reducing ICH mortality.

### Meta-analysis on safety

Seven trials reported incidences of adverse events (73, 76, 77, 84, 85, 88, 90). Only one trial reported three cases of skin rashes related to PNS injection (56). No severe side effects were reported in the other six trials.

### Quality assessment

The quality assessment of the 20 included trials was evaluated in accordance with Jadad’s scale. Four trials were assessed as high-quality trials (scoring 3–5 marks) (76, 80, 88, 89), and the rest were assessed as low-quality trials (scoring 1–2 marks) owing to poor description on randomization and blindness in the papers. All trials mentioned randomization and dropout rate but only three of them described randomization methods (76, 80, 88) and one trial mentioned single blinding in their methodological design (89).

### Funnel plots

**Figure 8:**
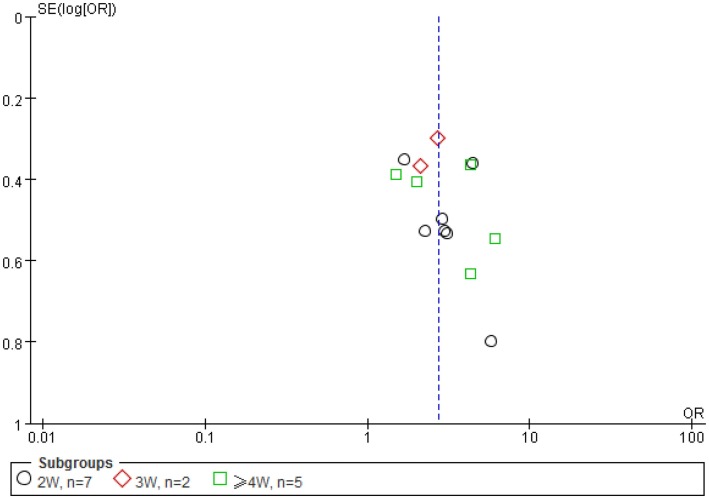
**A funnel plot was made using the data of effectiveness rate (ER)**. A total of 13 trials reported the effect rate, which was categorized into three subgroups by the evaluating time: (1) 7 trials assessed the ER at the end of 2 weeks of treatment, (2) 2 trials assessed the ER at the end of 3 weeks, and (3) 5 trials assessed the ER at the end of 4 weeks. Because of the small number of trials involved, the imperfect or asymmetrical funnel plot is not a reliable indication of potential publication bias in this case.

To determine potential publication bias, funnel plot based on the effective rate was elaborated (Figure 8). A total of 13 trials reported the effect rate, which was categorized into three subgroups by the treatment outcome evaluating time: (1) 7 trials assessed the ER at the end of 2 weeks of treatment, (2) 2 trials assessed the ER at the end of 3 weeks, and (3) 5 trials assessed the ER at the end of 4 weeks.

## Discussion

Intracerebral hemorrhage is the leading cause of death and disability in people with TBI and stroke. So far, no proven drug is available for ICH. From the meta-analysis of multiple clinical trials involving PNS treatment for intracerebral hemorrhage (ICH), we found that ICH patients treated with PNS exhibited better outcomes than ICH patients that received current treatments in all aspects examined including the ER, the NDS, intracranial hematoma volume (IHV), intracranial edema volume (IEV), BI, and NDP. This finding is consistent with the knowledge that *P. notoginseng* is an effective medical herb for wound healing and bleeding.

### Intervention time

Because intracerebral bleeding is a major cause of mortality and morbidity in ICH patients, timely intervention is critical for saving lives and for better outcomes of the patients. *P. notoginseng* is a hemostatic drug that has been used to stop bleeding after gun-shots and traumatic injuries since the Ming Dynasty (93). The non-protein amino acid dencichine and notoginsenoside Ft1 of PNS have been identified as the active hemostatic components of *P. notoginseng* (17).

The timing of PNS intervention varied among the 20 clinical trials examined. The earliest PNS intervention was given within 48 h of ICH onset (74, 80, 82, 84–86). These early treated patients had the following common characteristics: hemorrhaging of less than 30 ml, diagnosis of the precise bleeding site, no comas, and stable vital signs. The last PNS intervention began 3 weeks after ICH onset (73, 76). The majority of the patients, however, received PNS between 1 and 2 weeks after ICH onset. Only one trial examined the timing effect of PNS intervention on the outcome of ICH (88). In that trial, 60 ICH patients received 600 mg/day of PNS within 24 h after ICH onset, and 55 ICH patients received 600 mg/day of PNS injection 7 days after ICH onset. Both treatments lasted 14 days and both treatments improved the outcome of the ICH patients compared to the CT groups. No difference was found in the ER between the two PNS treatment groups (*P* > 0.5).

The results show that the death rate was two- to threefold lower in ICH patients that received PNS treatment within 48 h of ICH onset than those in the CT group (*P* = 0.05, Figure 6), suggesting early PNS intervention can be critical for saving the lives of ICH patients at risk of death.

Animal studies showed that PNS given at the third day of acute cerebral hemorrhage produced the best effects in reducing cerebral edema and hematoma volume in rats when compared to PNS administration given at other times (94). These authors reported up regulation of Bcl-2 expression in cerebral tissue after PNS treatment. Another animal model study showed that early PNS treatment after ICH onset can significantly suppress brain inflammation as reflected in reduced level of CAM-1 and TNF-α expression in PNS-treated rats (95). Thus early PNS intervention could minimize ICH-induced brain inflammation and neuronal apoptosis, and facilitate the restoration of normal brain function.

### Adverse events, dosage, and route of administration

Seven clinical trials included in this study reported the observation of adverse events (73, 76, 77, 84, 85, 88, 90). Only three cases of skin rash were reported after PNS (XST) injection in one trial (56), accounting for 5.5% of the 55 PNS-treated patients of that trial (Table 1). This finding is in agreement with our previous study that the side effects of PNS use are relatively rare, with skin rash being the most common side effect of PNS use, accounting for more than 52% of all its adverse reactions (96).

There were considerable variations in the daily dose and total dose of PNS administration among the 20 clinical trials included in this study, with the lowest dose at 140 mg/day, the highest dose at 800 mg/day, and the average dose at 373.25 ± 181.57 mg/day. Most of the trials administered PNS at a dose range between 300 and 400 mg/day, with 12 trials at a dose greater than 300 mg/day. So far, there is no consensus about the ideal daily dose and total dose of PNS for ICH although 200–300 mg/day for 2–4 weeks are usually recommended by the manufacturers of medical PNS.

For the treatment duration and the total dose, some studies recommend a treatment course of 14 days (78), but others propose a 30–40-day treatment regime for achieving a better outcome (74, 97). Of the 20 clinical trials included in this study, 1 trial treated the ICH patients for 1 week, 2 trials for 11 weeks, 4 trials for 3 weeks, 3 trials for 4 weeks, and 1 trial for 6 weeks (Table 1). Our analysis shows that an increased duration of PNS treatment is linked to a better outcome of the intracerebral hemotoma volume (Figure 4). Significant improvement in IHV was found only after 10 or more days of PNS treatment. It appears that a minimum of 2–4 weeks of treatment is required to achieve significant improvement in the outcome of patients with acute ICH. A longer treatment could bring additional benefit but with an additional medical cost.

### Mortality

Significantly reduced morbidity and mortality are the key indexes of successful treatment of intracerebral hemorrhage. Of the 20 clinical trials, 3 trials reported short-term mortality in ICH patients who received PNS treatment within 48 h after ICH onset (74, 84, 88) (Figure 7), and 4 trials reported short-term mortality in ICH patients who received PNS treatment 7 days after ICH onset (Figure 7). Both treatment regimens exhibited a more significantly reduced the death rate in the PNS group than in the CT group (by >2, *P* < 0.05, and >3-fold, *P* < 0.01, respectively). This is consistent with a recent report that certain herbal ingredients of traditional Chinese medicine (TCM) could stimulate the activation of blood, resolve hemostasis, and reduce acute ICH-induced short-term mortality (10).

### Quality of life

The quality of life of the ICH patients is closely associated with the severity of the disease and the efficacy of the treatment (98, 99) and is often assessed using the NDS and the BI. Of the 20 trials included in the present study (with a total of 1,891 ICH patients), only 2 trials measured BI (Figure 4) and reported improved activities of daily living in the PNS-treated ICH patients compared to the patients in the CT group.

Neurological deficit score is an important index for the diagnosis of symptom severity and functional recovery of the patients. Three of the trials included in this study reported NDS at 7, 15, 21, 28, and 30 days after PNS treatment (74, 77, 86) (Figure 2). The results showed that it took at least 21 days for the PNS treatment to produce noticeable improvements in ICH patients when compared with the CT groups (74, 77, 86). That improvement in terms of NDS, however, became significant at 28 days in one study (86) and 40 days in another (74) after the start of PNS treatment (*P* < 0.05, *P* < 0.01, respectively).

### Funnel plots

The funnel plot is often used to check for the existence of publication bias in meta-analyses, assuming that the largest studies will be plotted near the average, and smaller studies will be spread evenly on both sides of the average, creating a roughly funnel-shaped distribution. Deviation from this shape can indicate publication bias. Although the funnel plot appeared asymmetrical in this study, it may reflect the fact of unbalanced and very limited data of each group (2W *n* = 7, 3W, *n* = 2, 4W, *n* = 5) (Figure 8) used for the plot rather than a true publication bias as eight samples from each group is considered minimal (while more than 20 is preferred) number required for a meaningful funnel plot, and a less funnel plot may give a wrong impression of publication bias if high precision studies are different from low precision studies with respect to effect size (e.g., due to different populations examined) (100). The appearance of the funnel plot can also alter substantially depending on the scale on the *y*-axis (101).

### Mechanism of PNS action

Intracerebral hemorrhage can disrupt cerebral blood flow, energy metabolism, and the integrity of the blood–brain barrier (BBB), resulting in edema, inflammation, apoptosis, neurological dysfunction, and often death. Acute and chronic macro- and micro-bleeding and thrombosis are the primary determinants of hematoma and edema development. Agents that can effectively control the bleeding and reduce hematoma and edema hold the promise to become effective therapies for ICH and have been the major treatment targets of multiple international ongoing randomized control trials of ICH (102).

Our meta-analysis shows that total *P. notoginseng* saponin extract (PNS) could be a therapeutic agent for ICH because it can significantly attenuate edema and hematoma in ICH patients. This is in line with recent reports that PNS (XST) injection treatment (175 mg/day) for 2 weeks significantly improved hematoma absorption and neurological function in 32 acute ICH patients compared to 29 RT controls (103). PNS has also been shown to improve microcirculation around the hematoma ischemic area, promote the absorption of hematoma, slow down and inhibit brain edema development, and significantly shorten the time for the edema to disappear (104).

The mechanism of PNS’ neuroprotection against ICH injury remains to be fully understood. It may involve differential protective activities of various ginsenosides and notoginsenosides on hemostasis, anti-coagulant, anti-thrombotic, platelet aggregation and complement activation, hemorheology, blood viscosity and hematocrit, vasodilation, microcirculation, energy metabolism, oxidative stress, inflammation, and immune function. Some of the recently published PNS actions are presented below as they may be relevant or potential mechanisms of PNS in ICH.

### Hemostatic effects of PNS

Spontaneous acute and chronic macro- and micro-bleeding contribute directly to hematoma growth in TBI and ICH patients and are linked with symptom severity, recurrence, and poor outcome (105, 106). Preclinical TBI studies showed that the extent of ICH acquired during acute and subacute phases (3 h, 3, 9, and 23 days) post-ICH can predict the functional and histopathological outcome in rats 6–12 months later and is correlated with the final cortical atrophy (*P* < 0.05), hippocampal atrophy (*P* < 0.01), and memory deficits (*P* < 0.01) (107).

At least two PNS components, i.e., dencichine and notoginsenoside Ft1 have been identified to possess hemostatic properties that could block or minimize bleeding and hematoma expansion after ICH onset (17, 108). Dencichine is a bioactive non-protein therapeutic amino acid found in *P. notoginseng*. At low concentrations, dencichine has hemostatic and platelet-enhancing activity, but at high concentrations, it is neurotoxic (108). Decichine enhances hemostasis of activated platelets via AMPA receptors (109). Notoginsenoside Ft1 is a potent procoagulant that can induce dose-dependent and ADP-induced platelet aggregation, increase plasma coagulation indexes, decrease tail-bleeding time, and increase thrombogenesis and cytosolic Ca (2 +) accumulation. Dencichine and notoginsenoside Ft1 may underlie the hemostatic mechanism of PNS during the acute and subacute phases of ICH.

### Anti-thrombosis, fibrinolysis, and anti-coagulation mechanism: Role of nitric oxide

Patients with TBI and resultant intracranial hemorrhage (ICH) are at high risk for developing venous thromboembolism (VTE) (110). Intrahematomal blood clotting is also a pathogenetic factor in hyperacute perihematomal edema formation (111). There is an increasing use of anti-platelets and/or anti-coagulants in the treatment of blood clotting and hyperviscosity in ICH and there is some evidence of therapeutic effects in animal models of ICH (112–115). Several compounds of PNS including adenosine and guanosine, ginsenoside Rh1, F1, Rg1, and Rg2 have anti-platelet and anti-coagulant activities, with adenosine and guanosine and the ginsenosides as the main anti-platelet aggregation compounds of PNS (116–118).

One study showed that sanchinoside Rg1 markedly inhibited experimental thrombosis formation by enhancing the function of fibrinolysis system and stimulating vascular endothelial cells to release nitric oxide (NO) (119). Ginsenoside Rb1 can also reverse oxidative stress- and ischemia-related umbilical endothelial dysfunction and myocardial injury through upregulation of the endothelia NO synthase (eNOS) pathway in diabetes rat model (120, 121).

### PNS has anti-hypertension activity

Hypertenison is a critical pathological factor in triggering ICH onset. Notoginsenoside Ft1 activates both glucocorticoid and estrogen receptors to induce endothelium-dependent, NO-mediated relaxations in rat mesenteric arteries (122).

### PNS inhibits complement activation

PNS could improve the outcome of acute ICH by suppressing the complement 3 (C3)-mediated pathway. Activation of complement cascades plays an important role in anaphylatoxin-mediated inflammation, secondary toxicity, and brain damage after ICH (24, 123). Studies have shown that PNS (co-)therapy inhibited the enhancement of blood complement C3 levels in experimental ICH (86). Significant reduction in circulation complement (C3) was found in 43 rheumatoid arthritis patients treated with PNS for 28 days, and which was associated with improved clinical symptoms such as joint swelling index when compared to the control subjects (50). This could be a potential mechanism underlying the decreased volume of intracerebral edema in the patients receiving PNS treatment group reported in the three clinical trials (Figure 5). In ICH-induced local tissue inflammation, C3 promotes the adhesion, exudation and translocation of inflammatory cells, and stimulates the secretion of large amounts of inflammatory mediators such as TNF-α and IL-1β, resulting in an increased inflammatory response and brain damage. These responses are absent in mice deficient in C3 activity and show reduced inflammatory cell infiltration, brain edema formation, and improved neurologic outcome after experimental ICH (124, 125).

### PNS protects BBB integration

Blood–brain barrier disruption is a hallmark of ICH-induced brain injury and contributes to edema formation, the influx of leukocytes, and the entry of potentially neuroactive agents into the perihematomal brain, all of which can contribute to brain injury. Factors implicated in BBB disruption include: inflammatory mediators (e.g., cytokines and chemokines), thrombin, hemoglobin breakdown products, oxidative stress, complement proteins, and matrix metalloproteinases, etc. (126, 127). Two studies have shown that ginsenoside Rg1 provides neuroprotection against BBB disruption, edema formation, and neurological injury in rat models of cerebral ischemia/reperfusion through the downregulation of aquaporin 4 expression and anti-apoptosis pathways (38, 128).

### PNS protects against ischemia/reperfusion, and stimulates angiogenesis

Neurons are oxygen sensitive and are vulnerable to ischemic-reperfusion injury after ICH. Experimental studies have shown that PNS and ginsenosides Rb1 and Rb3 can provide significant protection against ischemia/reperfusion injury in rodent brains (36, 129), cardiomyocytes (130, 131), and kidneys (132). Ginsenoside Rb1 prevents homocysteine-induced endothelial dysfunction via PI3K/Akt activation and PKC inhibition (133). Ginsenoside Rg1 enhances angiogenesis after hypoxia ischemia brain damage in neonatal rats and in diabetic mice, in part through hypoxia-inducible factor (HIF-1a), glucocorticoid receptor (GR), and fibroblast growth factor receptor (VEGFR)-mediated pathways (134–138), and enhances the resistance of hematopoietic stem/progenitor cells to radiation-induced aging in mice (38). Notoginsenoside Ft1 promotes angiogenesis via HIF-1α mediated VEGF secretion and the regulation of PI3K/AKT and Raf/MEK/ERK signaling pathways (139). PNS also enhances VEGF signals and promotes angiogenesis derived from rat bone marrow and mesenchymal stem cells (140) as well as inhibit ischemia-induced apoptosis by activating the PI3K/Akt pathway in cardiomyocytes (141).

### PNS stimulates stem cell proliferation and differentiation

Cognitive impairment is common and is linked to neuronal cell loss after ICH (92). PNS could promote functional recovery of ICH patients through stimulating stem cell proliferation and differentiation. Studies have shown that ginsenoside Rb1 can improve spatial learning and memory by stimulating neurogenesis in the hippocampal subregions of rats (22) and that ginsenoside Rd can stimulate the proliferation of rat neural stem cells *in vivo* and *in vitro* (28). Ginsenoside Rg1 stimulates the proliferation and differentiation of human dental pulp stem cells and facilitates neural differentiation of mouse embryonic stem cells via the GR-dependent signaling pathway (31, 32), which promotes peripheral nerve regeneration in the rat model of nerve crush injury (142) and improves spatial learning-memory in dementia rats after bone marrow mesenchymal stem cell transplant (21). Ginsenoside Rg1 mediates microenvironment-dependent endothelial differentiation of human mesenchymal stem cells (143).

### PNS protects microcirculation from ischemia/reperfusion-induced injury

PNS treatment improved microcirculation around the hematoma ischemic area, promoted the absorption of hematoma, slowed down and inhibited brain edema development, and significantly shortened the time for the edema to disappear (104). Notoginsenoside R1 can attenuate ischemia/reperfusion (I/R)-induced microvascular hyperpermeability, inflammatory cytokine production, NF-kB activation, leukocyte rolling and adhesion, the expression of E-selectin in endothelium and CD18 in neutrophils, loss of tight junction proteins, and deficit in energy metabolism during I/R in rats (144, 145). Ginsenosides Rb1 and Rg1, and notoginsenoside R1 have been shown to protect lipopolysaccharide-induced microcirculatory disturbance in rat mesentery (146).

### PNS has ROS-scavenger, antioxidation, and anti-apoptosis properties

PNS has been shown to be a potent antioxidant in various experimental models. PNS induces thioredoxin-1 expression and prevents 1-methyl-4-phenylpyridinium ion-induced neurotoxicity (147). Ginsenoside Rb1 directly scavenges hydroxyl radicals and hypochlorous acid (103) and inhibits apoptosis in hydrogen peroxide-treated chondrocytes by stabilizing mitochondria and inhibiting Caspase-3 (148). Ginsenoside Rb1 also prevented MPP(+)-induced apoptosis in PC12 cells by activating estrogen receptors and ERK1/2/Akt pathways, and inhibiting SAPK/JNK/p38 MAPK pathways (149). Ginsenoside Rb1 protects against oxidative damage and renal interstitial fibrosis in rats with unilateral ureteral obstruction (150), and against beta-amyloid protein(1-42)-induced neurotoxicity in cortical neurons and in PC12 cells (30, 33) as well as against hypoxia and oxidative stress in rat retinal ganglion cells (29).

Ginsenoside Rd appears to be a superior neuroprotector with a wide therapeutic window in experimental stroke (151). Ginsenoside Rd attenuates redox imbalance, improves stroke outcome following focal cerebral ischemia in aged mice (152), and attenuates early oxidative damage and sequential inflammatory responses after transient focal ischemia in rats (153). Ginsenoside Rd prevents glutamate-induced apoptosis in rat cortical neurons (154), and promotes glutamate clearance by up-regulating the expression of glial glutamate transporter proteins (155). Ginsenoside-Rd exhibits anti-inflammatory activities through the enhancement of antioxidant enzyme activities and the inhibition of JNK and ERK activation *in vivo* (156).

Ginsenoside Rg1 protects against hydrogen peroxide-induced cell death in PC12 cells via the inhibition of NF-kB activation (157), reduction of nigral iron levels in MPTP-treated C57BL6 mice by regulation of iron transport proteins (158), and protection against beta-amyloid peptide-induced human endothelial cellapoptosis by activation of the GR-ERK signaling pathway (159). Oral Rg1 supplementation strengthens the antioxidant defense system against exercise-induced oxidative stress (160) and protects the liver against exhaustive exercise-induced oxidative stress in rats (161).

### PNS has anti-inflammation properties

Ginsenoside Rbl shows anti-neuroinflammation effects in rat models of Alzheimer’s disease (20) and prevents interleukin-1b-induced inflammation and apoptosis in human articular chondrocytes (162). Ginsenoside Rd inhibits the expression of iNOS and COX-2 by suppressing NF-kB in LPS-stimulated RAW264.7 cells and in mouse livers (163), and attenuates neuroinflammation in cultured dopaminergic neurons (164). Ginsenoside Re ameliorates inflammation by inhibiting the binding of lipopolysaccharides to TLR4 on macrophages (165). Ginsenoside Rg1 improves survival in a murine model of polymicrobial sepsis by suppressing the inflammatory response and apoptosis of lymphocytes (166). Ginsenoside Rg1 improves streptozocin (STZ)-induced diabetic nephropathy in rats by suppressing inflammatory reactions and expression of ectodermal dysplasia and TGF-beta (167). PNS also suppresses inflammation in a collagen-induced arthritis model (146).

### Anti-hyperglycemia and anti-hyperlipidemic effects of PNS

Hyperglycemia is associated with poor outcome in patients with TBI and ICH and in experimental models of ICH (93, 168, 169). PNS has hypolipidemic and antioxidant activities in rats with high-fat diets (170). Ginsenoside Rb1 has antiobesity and anti-hyperglycemic effects in rats (171). Ginsenoside Rb2 exerts its antidiabetic effects via activation of AMPK (172). Ginsenoside Rb2 lowers cholesterol and triacylglycerol levels in 3T3-L1 adipocytes under high cholesterol or fatty acids culture conditions (173). Ginsenoside Re reverses insulin resistance in muscles of high-fat diet rats (174). Ginsenoside Rg1 promotes glucose uptake through the activated AMPK pathway in insulin-resistant muscle cells (175).

### Many components and many mechanisms

Cerebral hemorrhage causes brain damage through multiple mechanisms, with spontaneous bleeding, hematoma development and perihematoma edema formation as the main factors contributing to the poor outcome of ICH. Effective therapies for ICH should be able to target all these factors. Our meta-analysis and literature review suggest that PNS is an effective therapy for acute ICH, and potentially functions through multiple mechanisms. Its most notable effects include hemostatic and anti-thrombotic effects, hemodynamic and hemorheological effects, angiogenesis and stem cell promoting effects, anti-hyperglycemia and anti-hyperlipidemia effects, and antioxidant and anti-inflammation effects, etc. Additionally, the strong tonic effects of PNS (176) could be beneficial to ICH patients, who are often weak and fragile during the recovery phase. A double-blind, double-dummy, randomized, and parallel-controlled study showed that 8 weeks after the onset of cerebral infarction, treatment of PNS tablets for 4 weeks significantly improved the outcome of the patients compared to the control treatments (177).

There are limitations to this research. Not all of the clinical trials analyzed were of high quality nor did they all include each desirable outcome evaluation. The number of ICH patients in each trial was often less than 100 and no long-term outcome data of the ICH patients were available. These and other factors should be controlled in further large scale studies so that the therapeutic effect of PNS can be better evaluated.

In conclusion, meta-analysis of the clinical trials suggests that PNS is superior to current treatment for acute ICH with minimal side effects. PNS could be an alternative therapy for acute ICH patients with a hemorrhagic volume of less than 30 ml. More clinical trials with better experimental designs could be conducted in the US and Europe to verify and extend the current findings and to determine the long-term effects of *P. notoginseng* on the recovery and recurrence of ICH patients.

## Conflict of Interest Statement

The authors declare that the research was conducted in the absence of any commercial or financial relationships that could be construed as a potential conflict of interest.

## Supplementary Material

The Supplementary Material for this article can be found online at http://www.frontiersin.org/Journal/10.3389/fneur.2014.00274/abstract
