# Supplementary material for: Efficacy and Safety of Panax notoginseng Saponin Therapy for Acute Intracerebral Hemorrhage, Meta-Analysis, and Mini Review of Potential Mechanisms of Action
Source: Front Neurol. 2015 Jan 7;5:274. doi: 10.3389/fneur.2014.00274 (PMC4288044; doi:10.3389/fneur.2014.00274)
Supplement: Supplementary file 1 [file DataSheet_1.PDF]

CBMdisc Search strategy:
1. Outcome assessment (healthcare) [expansion of all trees] /all subheadings
2. efficacy or treatment result or treatment outcome or therapeutic effect
3 clinical evaluation or clinical analysis or clinical comparison or clinical observation or clinical assessment
4. TT: (EFFICACY or EFFECT) and (CLINICAL or TREATMENT or THERAPEUTIC)
5. # 1 or # 2 or # 3 or # 4
6. Sanqi or notoginseng or notoginseng powder or notoginseng preparation or tianqi or tianqi notoginseng
7. Panax notoginseng saponins or Xueshuantong or Xuesetong or Passepartout or Luotai
8. # 6 or # 7
9. Sanqi / notoginseng [extension of all the trees] / All subheadings
10 # 8 or # 9
11 # 5 and # 10
12 # 11 and Example
13 randomized controlled trials [expanding all trees] / All subheadings
14 sample randomization [expanding all trees]
15 single-blind
16 double-blind
17 blind
18 triple-blind
19 placebo control
20 randomize
21 # 13 or # 14 or # 15 or # 16 or # 17 or # 18 or # 19 or # 20
22 # 21 and Examples
23 # 12 and # 22

Medline(OVID) search strategy:
1 RANDOMIZED-CONTROLLED-TRIAL in PT.mp. [mp=ti, ot, ab, nm, hw]
2 CONTROLLED-CLINICAL-TRIAL in PT.mp. [mp=ti, ot, ab, nm, hw]
3 RANDOMIZED-CONTROLLED-TRIALS.mp. [mp=ti, ot, ab, nm, hw]
4 RANDOM-ALLOCATION.mp. [mp=ti, ot, ab, nm, hw] (53641)
5 DOUBLE-BLIND-METHOD.mp. [mp=ti, ot, ab, nm, hw]
6 SINGLE-BLIND-METHOD.mp. [mp=ti, ot, ab, nm, hw]
7 1 or 2 or 3 or 4 or 5 or 6
8 TG=ANIMAL.mp. [mp=ti, ot, ab, nm, hw]
9 TG=HUMAN.mp. [mp=ti, ot, ab, nm, hw]
10 TG=ANIMAL.mp. [mp=ti, ot, ab, nm, hw]
11 ((TG=ANIMAL not TG=HUMAN) and TG=ANIMAL).mp. [mp=ti, ot, ab, nm, hw]
12 7 not 11
13 CLINICAL-TRIAL in PT.mp. [mp=ti, ot, ab, nm, hw]
14 explode CLINICAL-TRIALS.mp. [mp=ti, ot, ab, nm, hw] (0)
15 CLIN$.mp. [mp=ti, ot, ab, nm, hw]
16 TRIAL$.mp. [mp=ti, ot, ab, nm, hw]

17 (CLIN$ adj25 TRIAL$).ab. (98368)
18 CLIN$.mp. [mp=ti, ot, ab, nm, hw]
19 TRIAL$.mp. [mp=ti, ot, ab, nm, hw]
20 (CLIN$ adj25 TRIAL$).ti.
21 SINGL$.mp. [mp=ti, ot, ab, nm, hw]
22 DOUBL$.mp. [mp=ti, ot, ab, nm, hw]
23 TREBL$.mp. [mp=ti, ot, ab, nm, hw]
24 TRIPL$.mp. [mp=ti, ot, ab, nm, hw]
25 BLIND$.mp. [mp=ti, ot, ab, nm, hw]
26 MASK$.mp. [mp=ti, ot, ab, nm, hw]
27 ((SINGL$ or DOUBL$ or TREBL$ or TRIPL$) adj25 (BLIND$ or MASK$)).mp. [mp=ti, ot, ab, nm, hw]
28 (27 in TI or 27 in AB).mp. [mp=ti, ot, ab, nm, hw]
29 PLACEBOS.mp. [mp=ti, ot, ab, nm, hw]
30 PLACEBO$.mp. [mp=ti, ot, ab, nm, hw]
31 PLACEBO$ in TI.mp. [mp=ti, ot, ab, nm, hw]
32 PLACEBO$.mp. [mp=ti, ot, ab, nm, hw]
33 PLACEBO$ in AB.mp. [mp=ti, ot, ab, nm, hw]
34 RANDOM$.mp. [mp=ti, ot, ab, nm, hw]
35 RANDOM$ in TI.mp. [mp=ti, ot, ab, nm, hw]
36 RANDOM$.mp. [mp=ti, ot, ab, nm, hw]
37 RANDOM$ in AB.mp. [mp=ti, ot, ab, nm, hw]
38 REAEARCH-DESIGN.mp. [mp=ti, ot, ab, nm, hw]
39 13 or 14 or 17 or 20 or 28 or 29 or 31 or 33 or 35 or 37 or 38
40 TG=ANIMAL.mp. [mp=ti, ot, ab, nm, hw]
41 TG=ANIMAL.mp. [mp=ti, ot, ab, nm, hw]
42 TG=HUMAN.mp. [mp=ti, ot, ab, nm, hw]
43 ((TG=ANIMAL not TG=ANIMAL) and TG=HUMAN).mp. [mp=ti, ot, ab, nm, hw]
44 39 not 43
45 44 or 12
46 (sanqi or sanchi or panax notoginseng or xuesetong or xuesaitong or luotai or lulutong or sanqizongzaodai or naoming or xueshuantong).mp. [mp=ti, ot, ab, nm, hw]
47 45 and 46
48 from 47 keep 1-10

Manually retrieved Chinese magazines:
1 TCM magazine:
"Traditional Chinese Medicine", "Hunan Traditional Chinese Medicine", "Hubei Traditional Chinese Medicine", "Zhejiang Traditional Chinese Medicine," "Shanghai Traditional Chinese Medicine", "Shandong Traditional Chinese Medicine", "Jiangsu Chinese medicine", "Sichuan Traditional Chinese Medicine" "New TCM", "TMC for acute symptoms/emergency", "Hebei Traditional Chinese Medicine", "Henan Traditional Chinese Medicine", "Shaanxi Traditional Chinese Medicine", "Guangxi Traditional Chinese Medicine," "Fujian Traditional Chinese Medicine", "Journal of Information on Traditional Chinese Medicine."
2 Integrative TCM-Western Medicine magazines:

"Integrative Medicine Journal of Cerebrovascular Diseases Heart", "Chinese Journal of Integrative Medicine Emergency", "China Rehabilitation", "Chinese and Western Integrative Medicine", "Chinese Journal of Neurology", "China Journal of Neuropsychiatry "," Chinese Journal of Emergency Medicine "," Journal of TCM Internal Medicine, "" Stroke and Nervous Diseases. "

3 Chinese Materia Medica magazines:

"Formulated TCM medicine", "Chinese Materia Medica", "Chinese herbal medicine", " Chinese Medical Herbs", "Pharmacology and Clinics of Chinese Medical Herbs"
